# N-(1-carbamoyl-2-phenylethyl) butyramide reduces antibiotic-induced intestinal injury, innate immune activation and modulates microbiota composition

**DOI:** 10.1038/s41598-019-41295-x

**Published:** 2019-03-18

**Authors:** Adriano Lama, Chiara Annunziata, Lorena Coretti, Claudio Pirozzi, Francesca Di Guida, Allegra Nitrato Izzo, Claudia Cristiano, Maria Pina Mollica, Lorenzo Chiariotti, Alessandra Pelagalli, Francesca Lembo, Rosaria Meli, Giuseppina Mattace Raso

**Affiliations:** 1 Department of Pharmacy, University of Naples “Federico II”, Via D. Montesano 49, 80131 Naples, Italy; 2 Task Force on Microbiome Studies, University of Naples “Federico II”, Via D. Montesano 49, 80131 Naples, Italy; 3 Institute for Experimental Endocrinology and Oncology, IEOS, Via S. Pansini 5, 80131 Naples, Italy; 4 Department of Biology, University of Naples “Federico II”, Cupa Nuova Cinthia 21, 80126 Naples, Italy; 5 Department of Molecular Medicine and Medical Biotechnology, University of Naples “Federico II”, Via S. Pansini 5, 80131 Naples, Italy; 6 Department of Advanced Biomedical Sciences, University of Naples “Federico II”, Via S. Pansini 5, 80131 Naples, Italy; 7 Institute of Biostructure and Bioimaging, Consiglio Nazionale delle Ricerche CNR, Via S. Pansini 5, 80131 Naples, Italy

## Abstract

The use/misuse of antibiotics leads to pathological features referring to antibiotic-induced intestinal injury (AIJ), a clinical issue that plays a prominent role in the development of severe digestive disturbances. AIJ is characterized by loss of intestinal architecture and function, dysbiosis and bacterial translocation into the liver, triggering hepatic inflammation. This study aimed at determining the beneficial effect of N-(1-carbamoyl-2-phenylethyl) butyramide (FBA), a butyrate releasing compound, in ceftriaxone-induced intestinal injury. To this purpose, mice receiving ceftriaxone (8 g∙kg^−1^/die, per os) for five days, were treated with FBA (212,5 mg∙kg^−1^/die, per os) for five or fifteen days. FBA modulated key players of innate immunity in antibiotic-injured gut tissues, reducing inflammatory process and improving the anti-inflammatory and resolving pattern. FBA also improved colonic architecture and intestinal integrity. Interestingly, we also observed a remodeling of gut microbiota composition related to an increase of metabolic pathways related to lactate and butyrate production. At mechanistic level, FBA induced histone acetylation and increased the expression of GPR43 and monocarboxylate transporter 1 in colon. Our data clearly demonstrated that FBA has multiple converging mechanisms in limiting intestinal and hepatic alterations to counteract AIJ.

## Introduction

Antibiotics are one of the most used drug classes to manage infectious disorders. Although the discovery of antibiotics represents the breakthrough in medicine history, over the years, the medical overuse of broad spectrum agents has dramatically increased^1,2^ as well as the exposure to low levels of antibiotics contained in food and water^3^. Indeed, these drugs can induce several gastrointestinal (GI) adverse events, commonly ascribed to their ability of altering gut microbiota composition and diversity, the so-called dysbiosis. Epidemiological studies^4,5^ and data on antibiotic-induced alteration in gut microbiota indicate an increased risk of developing inflammatory bowel diseases and post-infective irritable bowel syndrome, secondary to dysbiosis and bacterial translocation^6,7^. Although there is great inter-individual variation in the composition of gut microbiota, a set of specified functions are shared among individuals referred to the activity of common core gut microbiome^8^, suggesting the importance of a defined consortium of gut bacteria and its related functions in maintaining host homeostasis. The mechanisms through which intestinal dysbiosis induced by antimicrobial drugs can trigger alterations at digestive and extra-digestive sites are poorly understood, and, most importantly, there is a lack of effective and specific therapeutic interventions for their management. AIJ is characterized by modifications of gut microbiota and intestinal architecture, with a shift from eubiosis to dysbiosis, leading to loss of beneficial metabolic activities of the colonic microbiota, i.e. extraction of nutrients, digestion of polysaccharides, energy metabolism, regulation of fat storage, synthesis of vitamins and short-chain fatty acids (SCFAs), essentials for gut functionality^9,10^. Among SCFAs, butyrate plays a pivotal role as nutrient for the colonic epithelium, influencing the composition of gut microbiota^11,12^ and modulating directly or indirectly immune and inflammatory response^13,14^. Butyrate carries out its activities through its interaction with G-protein coupled receptors, GCPRs, namely GPR41 and GPR43, and via inhibition of histone deacetylase (HDAC), modulating gene expression through an epigenetic mechanism^15^. Butyrate-induced GPR and downstream mitogen-activated protein kinase signaling activation, occurring through GPR41 and 43, regulates inflammatory pathways involved in gut health^16,17^. Moreover, activation of GCPRs by butyrate in the gut also produces the endocrine hormones glucagon-like peptide 1 (GLP-1) and peptide YY (PYY), involved in the control of glucose and lipid metabolism^18,19^. However, since the intracellular signaling pathways of butyrate are pleiotropic, the physiologic function and pharmacologic effects of butyrate are multivariate, depending upon tissue type, dosage and time effects.

Recently, we demonstrated that a butyrate derivative, N-(1-carbamoyl-2-phenylethyl) butyramide (FBA) improved inflammatory colitis induced by dextran sodium sulfate^20^. Indeed, FBA showed a pharmacological profile similar to that of butyrate, also in reducing steatosis^13^ and insulin resistance^21^ in high fat diet fed animals. Actually, the unpleasant taste and odour make extremely difficult the oral administration of butyrate, particularly in poorly compliant children. The aim of this study was to evaluate the protective and anti-inflammatory effect of FBA, owing better palatability and compliance than the parental drug, in colon tissue after AIJ by ceftriaxone exposure. We studied its capability in reducing gut inflammation, analyzing colonic cytokines, proteins involved in epithelium repair and integrity, colon responsiveness thorough TLRs expression, and liver innate response and in modulating gut microbiota composition, trying to obtain an overall picture on the early and late phases of antibiotic-induced injury and their pharmacological control by FBA. Finally, we tried to shed light on the mechanisms underlying FBA treatment, addressing the expression of proteins related to butyrate effects.

## Results

### FBA reduces colon inflammation and tissue damage

**Figure 1:**
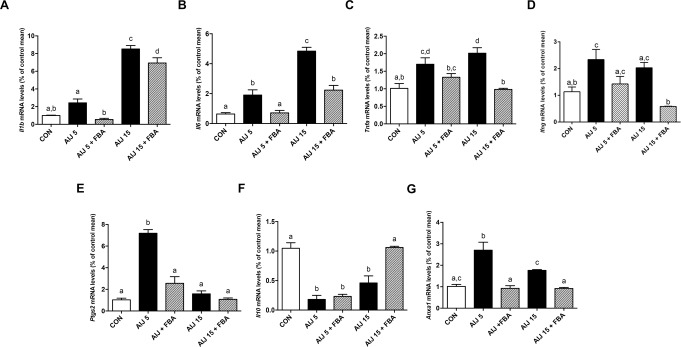
FBA reduces colon inflammation after 5 or 15 days of treatment. (**A**–**G)** Transcriptional levels of *Il1b*, *Il6*, *Tnfa*, *Ifng*, *Ptgs2*, *Il10*, *Anxa1* were evaluated in colonic tissues (*n* = 6). Real-Time PCR data are presented as mean ± S.E.M. All results were considered statistically significant at *P* < 0.05. Labeled means without a common letter differ, *P* < 0.05.

FBA modulated pro- and anti-inflammatory patterns in colon tissue. The mRNA expression of pro-inflammatory cytokines (IL-1β, IL-6, TNF-α, IFN-γ) and enzyme (COX-2) was widely enhanced in the colon of AIJ mice (Fig. 1A–E), while that of the anti-inflammatory cytokine IL-10 was reduced (Fig. 1F). FBA not only counteracted inflammation and improved anti-inflammatory response, but also normalized the expression of the resolvin annexin (Anx) A1 (Fig. 1G).

**Figure 2:**
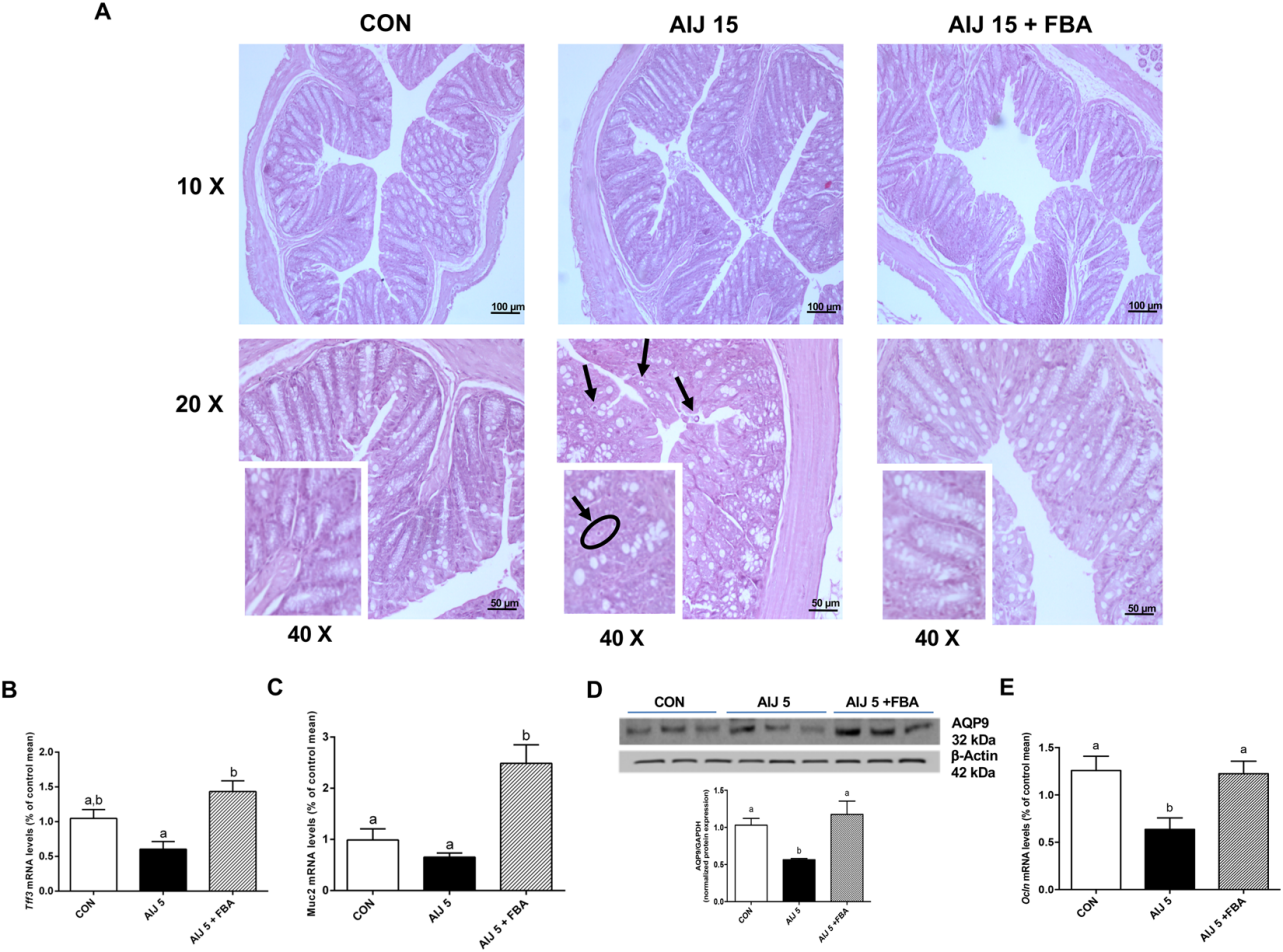
Effects of FBA on colonic morphology and integrity. (**A**) Distal colons from CON, AIJ 15, and AIJ 15 + FBA were stained with H&E. Scale bar: 100 µm (10x), and 50 (20x). Black arrows indicate apoptotic bodies. Representative pictures of all sections are shown. (**B**,**C**) The transcription of trefoil factor 3 (*Tff3*) and mucin 2 (*Muc2*) was also evaluated in colonic tissues of CON, AIJ 5 and AIJ 5 + FBA animals. **(D)** Representative immunoblot of AQP9 is shown (*n* = 6). The densitometric analysis of all determinations is also reported. All data are expressed as mean ± S.E.M. Equal loading was confirmed by β-actin. **(E)** mRNA expression of OCLN in colon tissue is also shown (*n* = 6). All results were considered statistically significant at *P* < 0.05. Labeled means without a common letter differ, *P* < 0.05.

Colon sections from AIJ 15 mice, stained with H&E, showed hyperplastic glands and apoptotic bodies (Fig. 2A, see arrows), with cell shrinkage, condensed cytoplasm and pyknotic and fragmented nuclei, compared to CON sections. Furthermore, the administration of ceftriaxone induced a reduced mucus thickness and an increased number of apoptotic bodies in colon sections (see Table 1). FBA treatment in injured animals normalized mucosa structure, showing a regular brush border and straight and unbranched crypts with lined largely goblet cells similar to those of CON (Fig. 2A). The improvement of mucus layer can be attributed to FBA capability in increasing the transcription of acute phase of protective trefoil factor 3 (TFF3) and mucin 2 (Fig. 2B,C).

**Table 1:** Histological scores of CON, AIJ and AIJ + FBA colon tissues at 15 day.

Histological parameters	CON	AIJ 15	AIJ 15 + FBA
Glandular hyperplasia	0-1 mitosis/HPF	1-2 mitosis/HPF	1-2 mitosis/HPF
Apoptosis	0-1/HPF	2-3/HPF	1-2/HPF
Mucus thickness	100	82,6	110,9

Score for glandular hyperplasia and apoptosis: 0 = none, 1 = mild, 2 = moderate, 3 = severe.

Score for mucus thickness (% vs control).

In the early phase of antibiotic-induced damage, AIJ 5 mice showed a significant reduction of AQP9, a water channel mainly located in the intestinal tract managing fecal water content^22^, and of occludin (OCLN), a crucial tight junction (TJ) for the maintenance of intestinal integrity (Fig. 2D,E).

### FBA reduces gut and liver inflammation, downregulating TLR4 and inhibiting NFκB activation

**Figure 3:**
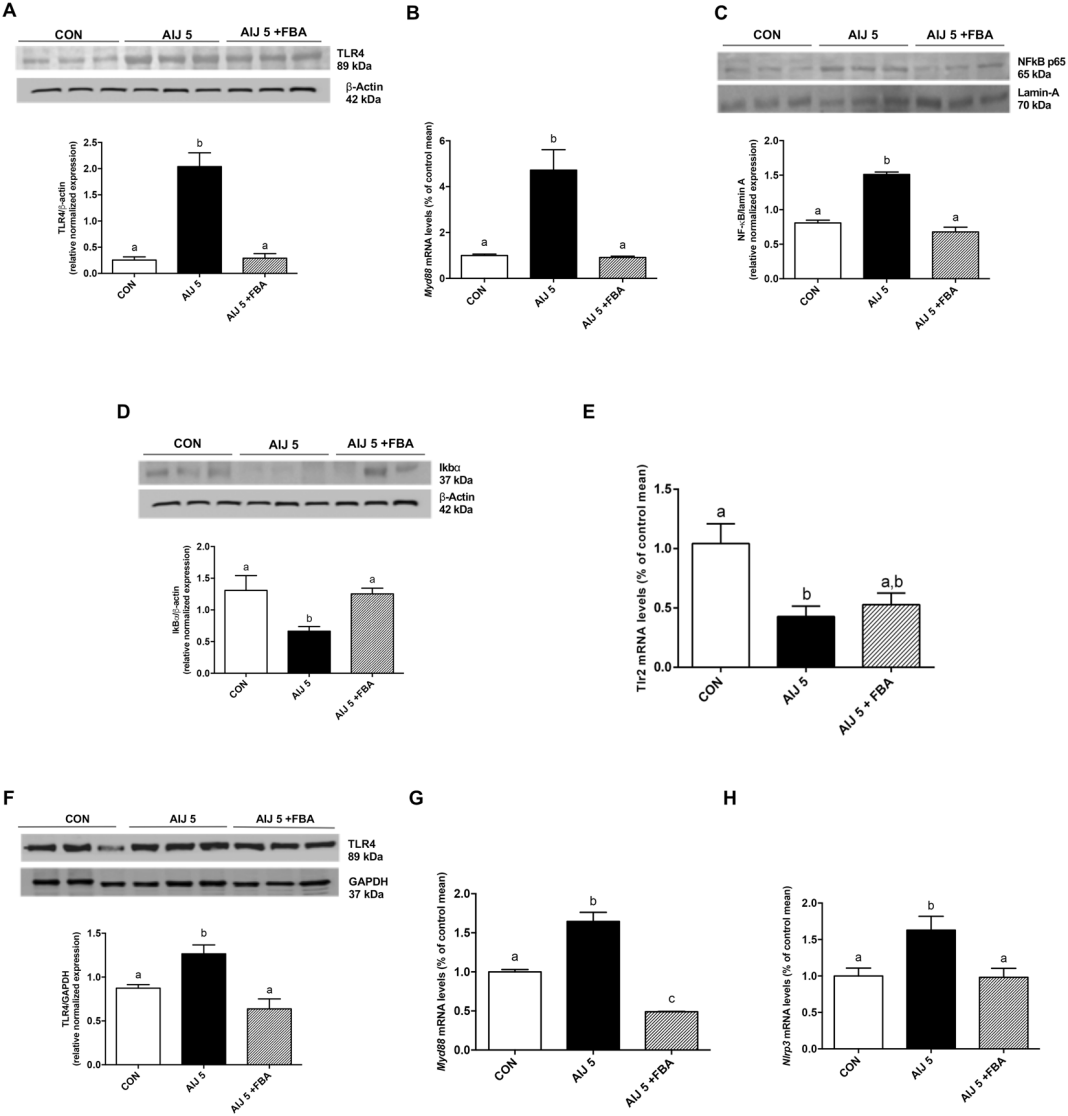
Effect of FBA on immunological and inflammatory mediators: gut-liver axis. (**A**–**D**) TLR4, Myd88, NFkB and Ikbα were determined in colonic tissues (*n* = 6). **(E)** mRNA expression of TLR2 in colon tissue is also shown. Furthermore, **(F**–**H)** the activity of FBA on TLR4, Myd88 and Nlrp3 was evaluated in liver (*n* = 6). For representative western blots, equal loading was confirmed by β-actin, lamin A or GAPDH. The densitometric analysis quantification of all determinations is also reported. All data are expressed as mean ± S.E.M. All results were considered statistically significant at *P* < 0.05. Labeled means without a common letter differ, *P* < 0.05.

As shown in Fig. 3A,B, after 5 d, antibiotic treatment increases TLR4 protein and Myeloid differentiation primary response (Myd) 88 mRNA expression in colon. FBA significantly inhibited these effects, and blocked the activation of NFκB pathway induced by ceftriaxone, preventing the translocation of NFκB p65 subunit in nucleus and consistently increasing IκB-α expression in cytosol (Fig. 3C,D). Antibiotic treatment also reduced TLR2 transcription, and a not significant increasing trend of its restoration was shown by FBA treatment (Fig. 3E). Interestingly, a similar profile of TLR4/Myd88 expression was observed in liver (Fig. 3F,G), probably due to a greater hepatic exposure to TLR ligands (i.e. Pathogen Associated Molecular Patterns, PAMPs, microbe-associated molecular patterns, MAMPs, and Damage-associated molecular patterns, DAMPs) deriving from the intestine. Liver inflammation was confirmed by the increase of mRNA transcription of inflammasome, NACHT, LRR and PYD domains-containing protein (NLRP) 3 (Fig. 3H) and limited by FBA treatment.

### Taxonomy-based analysis of microbiota changes

A subset of mice from each group was analyzed for gut microbiota by Illumina MiSeq technology. After processing, a total 1,514,762 valid reads were identified with an average of 63,115.08 ± 32,899.69 reads/sample and a sequencing depth of 23,422 reads/sample, representing the Good’s coverage of 99% or above for all samples (Table S1). All sequences were classified in 3,805 OTUs representing 18 phyla, 338 genera and 754 diverse species.

**Figure 4:**
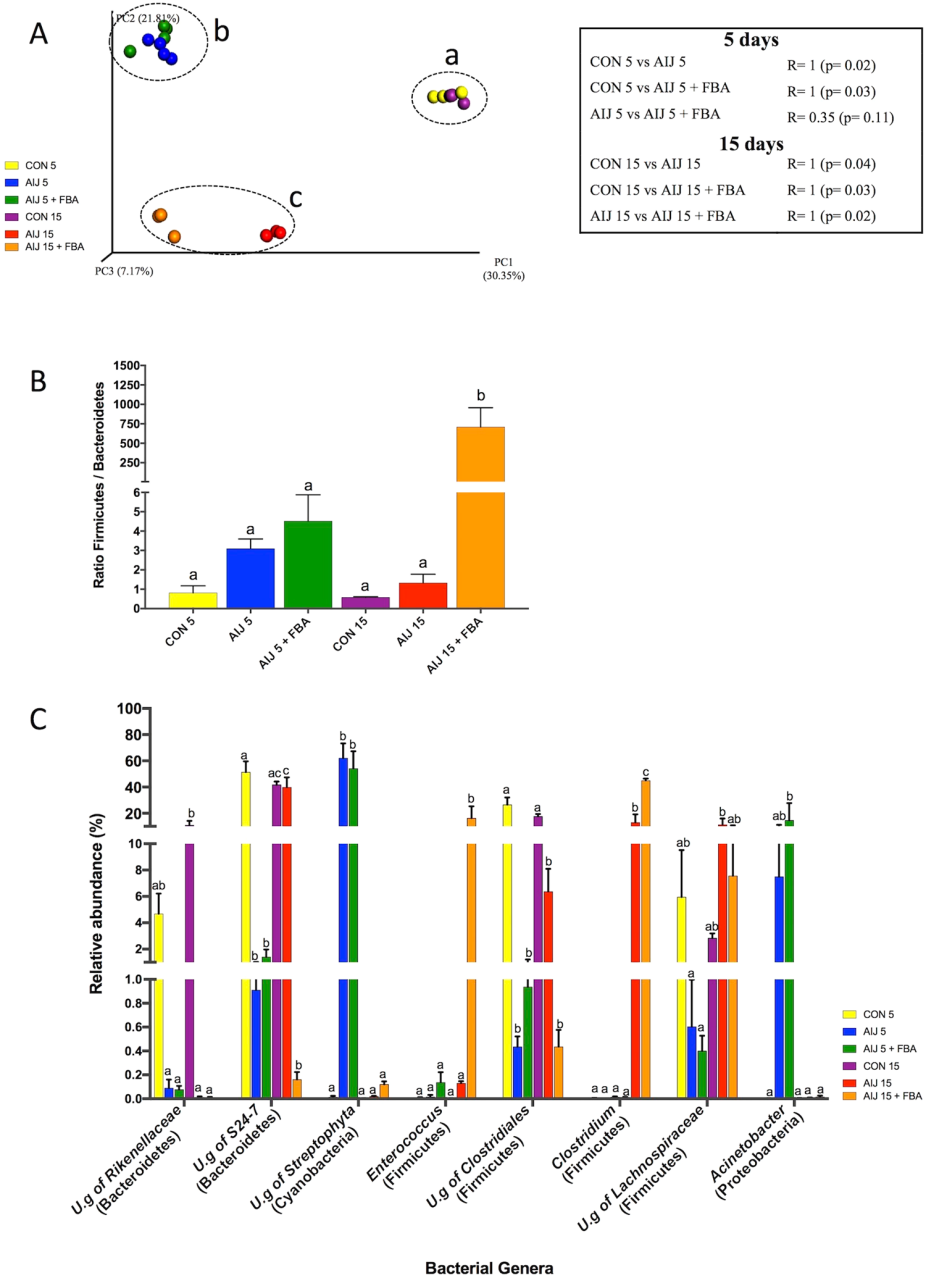
Taxonomy-based analysis of microbiota changes upon AIJ and FBA treatment at 5 and 15 days. (**A**) PCoA plot based on unweighted UniFrac distances of CON, AIJ and AIJ + FBA microbial communities at 5 and 15 days (23,422 sequences/sample). Analysis of similarity (ANOSIM) with 999 permutations was used to detect the statistical significant differences in microbial community composition among groups; on the right of the plot are reported both R statistics and p-values. (**B**) Ratio of Firmicutes and Bacteroidetes abundance in CON, AIJ and AIJ + FBA microbial communities at 5 and 15 days. (**C**) Percentage distribution of the bacterial genera with a mean relative abundance > 1% in at least one of the 6 groups found to be significantly different among CON, AIJ and AIJ + FBA microbial communities at 5 and 15 days. Variations in ratio and genera relative abundances among groups were assessed by one-way ANOVA followed by Tukey’s multiple comparison post-hoc test (different letters indicated significant differences among groups).

For alpha diversity analysis, Observed Species was significantly lower at AIJ 5 and AIJ 15 compared to CON groups, indicating a drastic decline in bacterial richness after antibiotic administration; upon FBA treatment at 5 and 15 days modification of species richness was observed in comparison with AIJ mice (Table 2). Phylogenetic distances of bacterial species among groups were measured by unweighted UniFrac analysis and reported in the principal coordinate analysis (PCoA) plot (Fig. 4A). In the plot, all controls (a), 5 day FBA treatment (b), and both 15 day AIJ groups in absence (c) or presence (d) of FBA showed non-overlapping clustering, indicating differences in microbiota structure among groups. Notably, at 15 days after AIJ, remodeling of microbiota structure was significantly different (R = 1; p = 0.02) in absence or presence of FBA (Fig. 4A).

**Table 2:** Species classification of key genera discriminating CON, AIJ and AIJ + FBA microbial communities at 5 and 15 days.

Genus	Species	CON 5	AIJ 5	AIJ 5 + FBA	CON 15	AIJ 15	AIJ 15 + FBA
*U.g of Rikenellaceae*	*Alistipes finegoldii*	1E-3 ± 1E-3^a^	0^a^	0^a^	1.93 ± 0.99^b^	1E-3 ± 1E-3^a^	2E-3 ± 1E-3^a^
	*Alistipes massiliensis*	1.36 ± 0.42^a^	0.01 ± 0.01^a^	0.03 ± 0.01^a^	4.17 ± 0.78^b^	4E-3 ± 2E-3^a^	5E-3 ± 3E-3^a^
	*Alistipes putredinis*	2.4 ± 1.45^a^	0.01 ± 0.01^b^	0.04 ± 0.01^b^	2.36 ± 0.66^a^	1E-3 ± 1E-3^b^	2E-3 ± 1E-3^b^
	*U.s. of Alistipes*	0.53 ± 0.32^a^	0.01 ± 0.01^a^	2E-3 ± 1E-3^a^	1.93 ± 0.84^b^	0^a^	0^a^
*U.g of S24-7*	*Barnesiella intestinihominis*	48.62 ± 8.15^a^	0.85 ± 0.1^b^	1.37 ± 0.55^b^	40.3 ± 2.15^c^	39.81 ± 7.42^c^	0.16 ± 0.06^b^
*U.g. of Streptophyta*	*Unclassified*	0.01 ± 0.01^a^	54.89 ± 10.48^b^	51.35 ± 12.49^b^	2E-3 ± 1E-3^a^	0.02 ± 3E-3^a^	0.08 ± 0.01^a^
*Enterococcus*	*U.s. of Enterococcus*	0^a^	0^a^	1E-3 ± 1E-3^a^	1E-3 ± 1E-3^a^	0.07 ± 0.02^a^	9.36 ± 5.03^b^
	*Enterococcus faecium*	6E-3 ± 6E-3^a^	0^a^	6E-3 ± 4E-3^a^	1E-3 ± 1E-3^a^	0.06 ± 0.01^a^	6.66 ± 3.75^b^
*U.g. of Clostridiales*	*Acetatifactor muris*	0.81 ± 0.5^a^	0.02 ± 0.01^bc^	0.05 ± 0.05^abc^	0.33 ± 0.01^abc^	0.01 ± 3E-3^bc^	1E-3 ± 1E-3^bc^
	*Alkaliphilus crotonatoxidans*	1.12 ± 0.33^a^	0^b^	0.04 ± 0.02^b^	0.43 ± 0.02^ab^	0^b^	1E-3 ± 1E-3^b^
	*Unclassified*	3.56 ± 3.08^a^	0.02 ± 0.02^b^	0.38 ± 0.14^b^	6.28 ± 1.7^c^	0.07 ± 0.03^b^	0.03 ± 0.01^b^
	*U.s. of Clostridium*	7.04 ± 2.57^a^	6E-3 ± 1E-3^b^	0.02 ± 0.02^b^	4.38 ± 0.12^c^	0.92 ± 0.45^d^	0.16 ± 0.06^b^
	*Anaerosporobacter mobilis*	0.03 ± 0.03^a^	0^a^	0^a^	0.89 ± 0.43^b^	4E-3 ± 4E-3^a^	0^a^
	*Clostridium aldenense*	2.53 ± 0.99^a^	0.11 ± 0.08^b^	0.01 ± 0.01^b^	1.45 ± 0.53^c^	0.13 ± 0.01^b^	1E-3 ± 1E-3^b^
	*Clostridium aminovalericum*	1.01 ± 0.98^a^	1E-3 ± 1E-3^b^	0^b^	0.13 ± 0.041^b^	0.01 ± 4E-3^b^	0^b^
	*Clostridium asparagiforme*	1.77 ± 0.56^a^	0.09 ± 0.04^b^	0.05 ± 0.02^b^	0.81 ± 0.03^c^	1.93 ± 1.04^a^	0.01 ± 4E-3^b^
	*Clostridium celerecrescens*	2.84 ± 2.54^a^	1E-3 ± 1E-3^b^	0.01 ± 0.01^b^	0.21 ± 0.07^b^	0.25 ± 0.13^b^	1E-3 ± 1E-3^b^
	*Clostridium lactatifermentans*	0.04 ± 0.01^a^	0.01 ± 0.01^a^	0^a^	0.1 ± 0.01^a^	1.01 ± 0.24^b^	0.14 ± 0.07^a^
	*Clostridium saccharolyticum*	1.84 ± 0.99^a^	2E-3 ± 2E-3^b^	5E-3 ± 3E-3^b^	0.64 ± 0.09^bc^	0.92 ± 0.53^c^	0^b^
*Clostridium*	*Clostridium butyricum*	0^a^	0^a^	0^a^	0.01 ± 4E-3^a^	12.75 ± 6.11^b^	38.95 ± 3.5^c^
	*Clostridium quinii*	0^a^	0^a^	0^a^	0^a^	0.04 ± 0.02^a^	5.25 ± 2.84^b^
*U.g. of Lachnospiraceae*	*Clostridium aldenense*	0.02 ± 0.01^a^	0^a^	1E-3 ± 1E-3^a^	6E-3 ± 3E-3^a^	8.83 ± 4.99^b^	3E-3 ± 2E-3^a^
	*Clostridium clostridioforme*	3.37 ± 2.74^a^	0^b^	0^b^	0.93 ± 0.53^b^	6E-3 ± 3E-3^b^	0.01 ± 0.01^b^
	*Clostridium hathewayi*	7E-3 ± 4E-3^a^	0^a^	1E-3 ± 1E-3^a^	2E-3 ± 1E-3^a^	0.3 ± 0.1^a^	5.97 ± 3.41^b^
*Acinetobacter*	*Unclassified*	0^a^	4.1 ± 1.88^a^	9.17 ± 8.46^b^	1E-3 ± 1E-3^a^	1E-3 ± 1E-3^a^	0.01 ± 0.01^a^

Reported is mean relative abundance ± S.E.M. of bacterial species according to SPINGO classification with significant differences (*P* < 0.05) among groups as assessed by one-way ANOVA followed by Tukey’s multiple comparison *post-hoc* test (different letters indicated significant differences among groups).

Comparison of gut microbiota in the different groups was carried out at phylum and genus levels (Table S2 and Fig. 4B). Reduction of Bacteroidetes and Firmicutes phyla and increase of Cyanobacteria and Proteobacteria were observed in AIJ 5 groups both in absence or presence of FBA (Table S2). After 15 days from antibiotic administration, the relative abundance of the described phyla was almost completely resumed to initial values; notably in FBA treated group the ratio of Bacteroidetes to Firmicutes was markedly shifted to Firmicutes (Fig. 4B). In particular, Firmicutes significantly increased in AIJ 15 + FBA compared to AIJ 15 (90.0% ± 5.80% vs 43.28% ± 7.10%, respectively), while Bacteroidetes significantly decreased (0.19% ± 0.06% vs 39.83% ± 7.43% in AIJ 15 + FBA and AIJ 15, respectively). Several genera differed significantly in abundance among groups (Fig. 4C). In particular, among genera with a mean relative abundance >1% our analysis showed a reduction of *U.g. of Rikenellaceae* and *S24-7* (Bacteroidetes) and of *U.g. of Clostridiales* and *Lachnospiraceae* (Firmicutes) and an increase of *U.g. Streptophyta* (Cyanobacteria) and *Acinetobacter* (Proteobacteria) in AIJ 5 groups both in absence or presence of FBA (Fig. 4C). After 15 days both in absence or presence of FBA, *U.g. Streptophyta*, *U.g. Lachnospiraceae* and *Acinetobacter* levels were reestablished, this did not occur for *U.g. of Rikenellaceae* and *U.g. of Clostridiales*. Among genera, FBA at 15 days increased of Firmicutes/Bacteroidetes ratio, mainly correlated to a decrease of *U.g. S24-7* (*Barnesiella intestinihominis*, phylum Bacteroidetes) and increase of *Enterococcus* (*E. faecium* and *U.s. of Enterococcus*, phylum Firmicutes) and *Clostridium* (*C. butyricum* and *C. quinii*, phylum Firmicutes) levels (Fig. 4C and Table 2). All bacterial species belonging to key genera are reported in Table S3, discriminating CON, AIJ and AIJ + FBA groups at 5 and 15 days.

**Figure 5:**
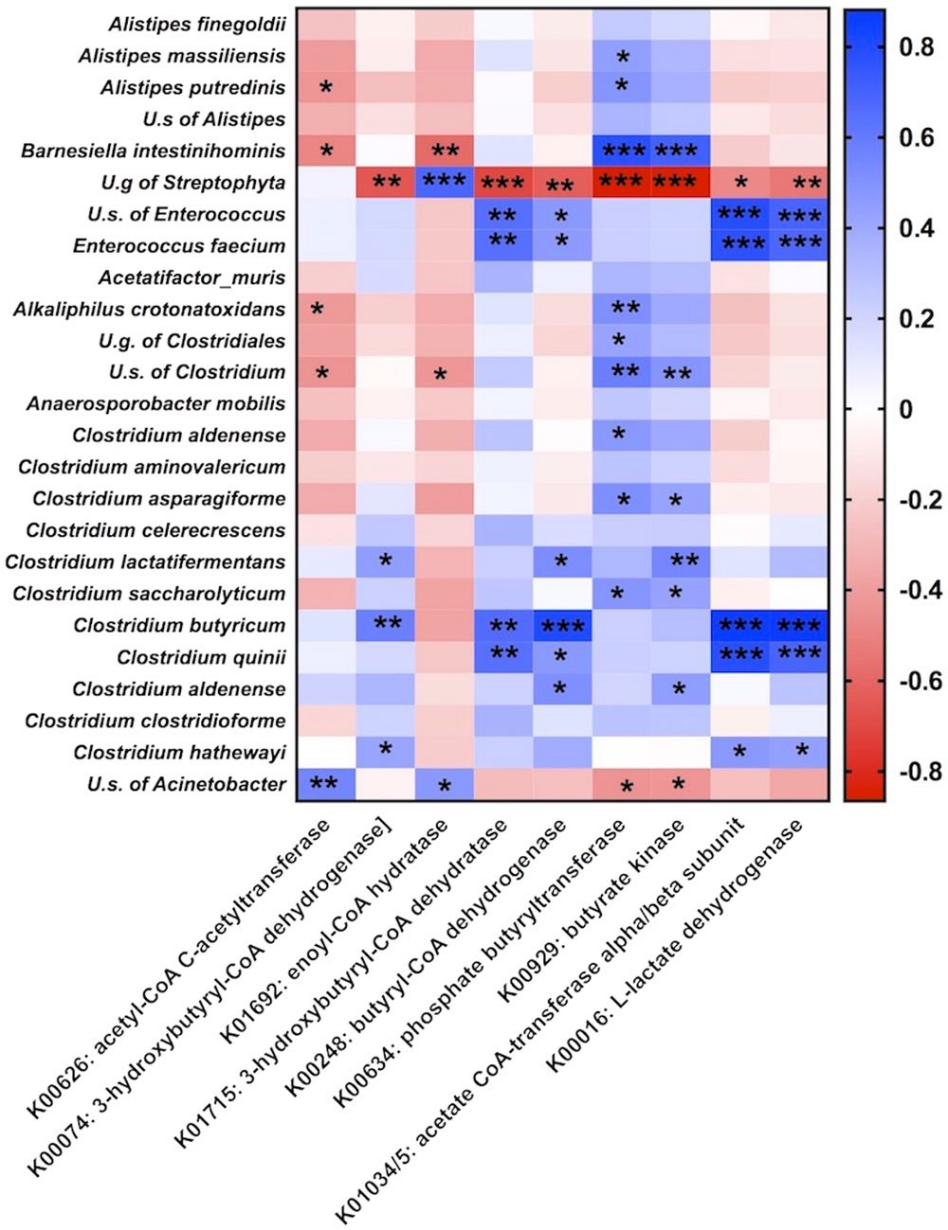
Heatmap showing the Pearson’s correlation between key functions counts (x-axis) and key species discriminating CON, AIJ and AIJ + FBA microbial communities at 5 and 15 days (y-axis). Blue or red colors designate the positive or negative correlations, respectively. *P < 0.05, **P < 0.01, ***P < 0.001.

To translate microbiota shift observed upon FBA administration into specific metabolic features of the corresponding microbiome, Phylogenetic Investigation of Communities by Reconstitution of Unobserved State (PICRUSt) analysis was applied. Considering butyrate producer assortment in clostridial species among groups, we focused on KEGG orthologs involved in butyrate metabolism (Table S3). The analysis showed significant enrichment of a key enzyme involved in butyrate metabolism (K01034/5) in AIJ 15 + FBA group compared to respective AIJ and CON groups. Furthermore, in AIJ 15, FBA administration induced a significant enrichment of K00016 function (L-lactate dehydrogenase), the enzyme catalyzing the interconversion of pyruvate and lactate (Table S3). Pearson correlation coefficient was used to associate PICRUSt data with identified key species discriminating CON, AIJ and AIJ + FBA microbial communities at 5 and 15 days (Fig. 5). Interestingly, data obtained showed a marked positive correlation among species belonging to genus *Enterococcus*, *C. butyricum* and *C. quinii* and butyryl-CoA:acetate CoA-transferase (K01034/5) and L-lactate dehydrogenase (K00016).

### The effects of FBA involve monocarboxylate transporter (MCT) 1, GPR43 and inhibition of HDAC

**Figure 6:**
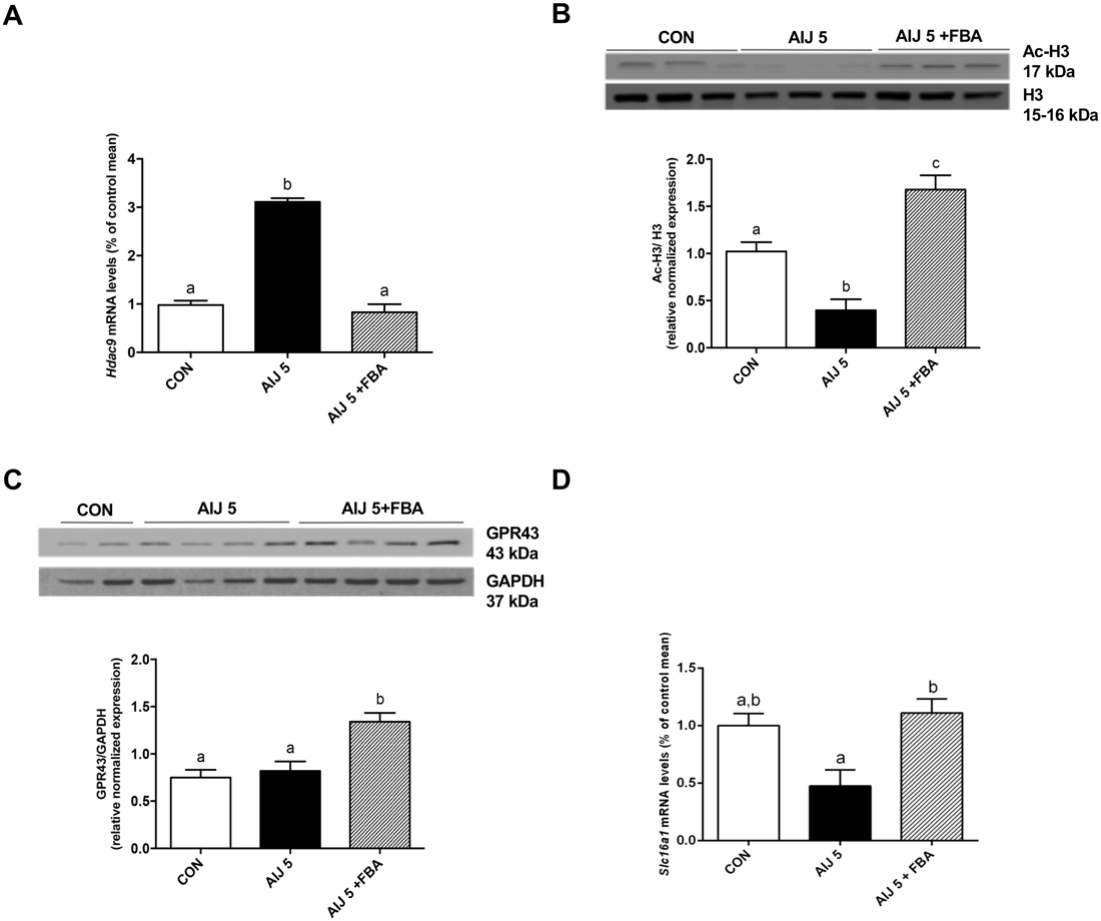
Mechanisms of anti-inflammatory activity mediated by FBA. (**A**,**B**) The butyrate derivative compound decreased transcriptional levels of Hdac9 and enhanced histone H3 acetylation (*n* = 6). Furthermore, (**C,D**) FBA increased protein level of butyrate receptor, GPR43 and mRNA expression of MCT1 (*n* = 6). For representative western blots, equal loading was confirmed by H3 and GAPDH. The densitometric analysis quantification of all determinations is also reported. All data are expressed as mean ± S.E.M. All results were considered statistically significant at *P* < 0.05. Labeled means without a common letter differ, *P* < 0.05.

FBA, as butyrate-releasing derivative, epigenetically regulates gene expression, inhibiting the mRNA expression of *Hdac9* and increasing the acetylation of nuclear histone H3 in colon, both altered by ceftriaxone challenge (Fig. 6A,B). Previous data showed a reduction of the solute transporter of butyrate MCT1 expression and function in intestinal inflammation^23^. FBA exerts its beneficial effects, increasing GPR43 protein and restoring solute carrier family 16 member *(Slc16a) 1* transcription, partially altered in AIJ 5 (Fig. 6C,D).

## Discussion

In this study, we demonstrated the efficacy of FBA, a butyrate-releasing compound, in limiting intestinal injury induced by oral administration of ceftriaxone and its capability in remodeling gut microbiota. Here, we provide a comprehensive picture of gut perturbations in terms of serum (see Fig. S1) and colon inflammation, liver alteration and microbial dysbiosis and the modulating effect of FBA on all these pathological features consistently associated to antibiotic exposure. Many studies mainly focused on antibiotic impact on gut microbiota composition, overlooking the detrimental effects on inflammation-induced damage associated to dysbiosis, causing low drug tolerability and patient noncompliance.

Here, we have characterized the intestinal injury induced in a dysbacteriosis model by oral administration of ceftriaxone^24,25^, underlying the time-dependent (5 and 15 days) alteration of intestinal homeostasis, the development of inflammatory damage and the impact on gut microbiota composition and diversity. Indeed, we have shown ceftriaxone-induced intestinal inflammation at 5 d, that was even more marked at 15 d, ten days after antibiotic withdrawal. The unbalance of pro- and anti-inflammatory mediators by ceftriaxone has been associated to an alteration of occludin transcription in colonic mucosa, indicating an impairment of its integrity^25^. The disrupted organization of mucosa increased the risk of bacteria endotoxin translocation^26^, confirmed by the increase of circulatory pro-inflammatory cytokines, suggesting that intestinal inflammation affected systemic immunity. Actually, FBA showed an anti-inflammatory effect even in the early phase of AIJ, reducing colonic expression of cytokines, lasting for 15 days, as also evidenced by histological analysis by an improvement of mucosa structure characterized by regular brush border and crypts and reduced presence of apoptotic bodies.

TJ proteins contribute to the primary physical barrier in intestine and the loss of their expression is associated to several inflammatory bowel diseases^27^. We have recently demonstrated that FBA, as well as sodium butyrate, restored gut integrity in mice with dextran sulfate sodium–induced colitis^20^. Consistently, in ceftriaxone-induced damage, FBA treatment restored the transcription of OCLN, counteracting the leaky gut, and increased AQP9 expression involved in the restoration of water and solute homeostasis^22^. Previous findings clearly indicated the involvement of AQP9 in the synthesis and secretion of mucus, which protects the intestinal surface and improves the flow of the intestinal content^28^. Indeed, apart from a restoration of mucus thickness, shown by histological analysis at 15d, FBA was able to modulate, already at 5-day treatment, MUC2 expression, whose increase together with that of TFF3, strengthened the reparative effect of FBA. The altered intestinal integrity leads to translocation of PAMPs and DAMPs into the liver, triggering a vicious loop between gut and liver and contributing to systemic inflammation^29^. Indeed, ceftriaxone not only increased TLR4/MyD88 pattern in the colon, but also in the liver, where NLRP3 appeared up-regulated, suggesting the activation of innate immune response and hence inflammation. FBA prevented immune response activation, both in colon and liver, damping inflammasome upregulation and NFκB activation. This latter event in turn induces the expression of pro-inflammatory cytokines, that initialize Th1 cells and promote a vicious self-feeding cycle. While TLR4 senses lipopolysaccharide from gram-negative, initiating its signaling, TLR2 recognizes gram-positive bacteria, indicating TLR2 and TLR4 role in intestinal homeostasis. Notably, colonic TLR2 appeared down-regulated in AIJ mice, an expression profile that was already shown by Grasa *et al*.^30^ 7 days after antibiotic treatment of mice. This data suggested an alteration at colonic level in the recognition of gram-positive and -negative bacteria after antibiotic administration, a pathological alteration partially reverted by FBA treatment.

The protective effect of FBA was confirmed by the decrease in serum AST and ALT, highlighting that the reduction of intestinal inflammation can reduce liver alteration and normalize immune tolerance. These data are consistent with our previous studies on FBA anti-inflammatory effect both in gastrointestinal and extra-intestinal disorders^13,14,21^.

Furthermore, by deep sequencing approach, we evaluated the modulation of gut microbiota composition upon oral administration of ceftriaxone and FBA. Antibiotic treatment induced severe modifications of gut microbiota, including: strong depletion of bacterial richness, marked increase of Proteobacteria and Cyanobacteria generally represented at low levels in healthy gut, and reduction of Bacteroidetes and Firmicutes phyla, including butyrate-producing bacteria. The detriment of butyrate-producing bacteria may impact gut homeostasis, since butyrate is the major source of energy for colonocytes, defending the host from inflammation and cell apoptosis^31^. The main altered microbial taxa were mostly restored to early values at 15 days, ten days after antibiotic withdrawal, indicating a trend toward reestablishing typical balance of gut microbiota. Fifteen days of FBA treatment in AIJ mice powerfully increased colonization of Firmicutes, and specifically of *Enterococcus* (*E. faecium* and *U.s. of Enterococcus*) and *Clostridium* (*C. butyricum* and *C. quinii*) genera. *C. butyricum* is a probiotic strain promoting the proliferation of beneficial bacteria, such as *Lactobacillus* and *Bifidobacterium* in intestinal disorders treatment^32^. Ling *et al*.^24^, in a model of antibiotic-associated diarrhea induced by ceftriaxone administration, have shown that a mixture of *C. butyricum* and *B. infantis* induces beneficial effects on the restoration of the intestinal microbiota and the recovery of the tissue architecture. Studies revealed the role of *Enterococcus spp*., Lactobacillales order, in human health as conflicting; *Enterococcus* may be an opportunistic pathogen under certain circumstances, as reported for colorectal cancer patients, and an early colonizer of gut microbiota in physiological condition exhibiting probiotic properties^33,34^.

FBA administration markedly influenced gut microbiota composition and potentially its functions. Correlation analysis showed a marked positive association between key species and specific enzymes, namely butyrate producers (*C. butyricum*, *C. quinii)* and lactate producers (*E. faecium)* with butyryl-CoA:acetate CoA-transferase and L-lactate dehydrogenase KEGG functions. Our results suggest that FBA treatment positively modulates metabolic pathways aimed to enhance lactate and butyrate production and select definite *Enterococcus* and *Clostridium* species that can operate metabolic cross-feeding.

Many butyrate effects are mainly mediated by two mechanisms: the epigenetic modulation by direct HDAC inhibition, causing hyperacetylation of histones^35^ or/and the direct interaction with its receptors, GPR41 and GPR43^36^. As previously demonstrated, butyrate upregulates the histone H3 acetylation of Foxp3 and promotes the differentiation of Treg, that may contribute to its anti-inflammatory activity^37^. Furthermore, HDAC9 normally suppresses Treg activation, therefore HDAC9 modulation, regulating the expression of heat shock protein 70 and controlling Foxp3, may be considered a new therapeutic target to limit colonic inflammation^38^. Consistently, in our experimental condition, FBA inhibited HDAC9 and induced the H3 acetylation.

Here, we also demonstrated that FBA induced the expression of butyrate receptor, GPR43. Indeed, several data indicated that SCFAs, including butyrate, directly and indirectly regulate colonic T cells differentiation, activating GPR43^39^. Downregulation of MCT1 expression and/or activity has been evidenced in inflammation of colonic mucosa^40^, colon cancer^41^, and in response to enteric infection and diarrhea caused by enteropathogenic *E. coli*^42^. Moreover, IFNγ and TNF-α signaling down-regulates MCT1 expression during inflammation, leading to butyrate deficiency in intestinal epithelial cells^43^. On the other hand, our and other studies showed an upregulation of MCT1 expression by colon tissues in response to butyrate administration^20,44^. Here, FBA induced the transcription of butyrate transporter, increasing cellular butyrate availability and thus possibly contributing to the recover the intestinal inflammatory damage induced by ceftriaxone.

All these data evidence that FBA carried out its pharmacological activity through multiple mechanisms, converging on inflammation control and resolution and modulation of microbiota composition, supporting its potential in AIJ.

## Material and Methods

### Animals and Experimental Design

The intestinal dysbiosis was induced in C57Bl/6 male mice (10-weeks-old, 25 ± 2 g, Harlan-Corezzano, Italy) by oral administration of ceftriaxone sodium (8 g∙kg^−1^ dissolved in water, Rocephin, Roche S.p.A., Milan, Italy) once daily for 5 days. Antibiotic-treated mice (AIJ) were treated simultaneously with FBA (212,5 mg∙kg^−1^ per die, dissolved in water, per os, once daily) for 5 days or until 15^th^ day, in order to evaluate its time-dependent effect.

Mice were randomly divided into the following five groups (n = 10, each group): 1) control (CON) mice receiving water by gavage as vehicle; 2) mice receiving ceftriaxone for five days (AIJ 5); 3) AIJ 5 mice receiving at same time FBA (AIJ 5 + FBA); 4) mice receiving ceftriaxone for five days and sacrificed at day 15 (AIJ 15); 5) AIJ 15 mice receiving FBA for 15 days (AIJ 15 + FBA). All procedures involving animals and their care were conducted in conformity with international and national law and policies (EU Directive 2010/63/EU for animal experiments, ARRIVE guidelines and the Basel declaration including the 3 R concept). The reported procedures were approved by the Institutional Committee on the Ethics of Animal Experiments (CSV) of the University of Naples Federico II and by the Ministero della Salute under protocol no. 886/2015-PR. As suggested by the animal welfare protocol, all efforts were made to minimize animal suffering, reducing the number of animals necessary to produce reliable scientific data.

### RNA extraction and Real-time semi-quantitative PCR

Mice colons were dissected at 5^th^ and 15^th^ day of the experimental period. After wash with PBS to remove the presence of feces, colons were immediately frozen in liquid nitrogen. Total RNA was extracted using TRIzol Reagent (Bio-Rad Laboratories) and following a specific RNA extraction kit (NucleoSpin®, MACHEREY-NAGEL GmbH & Co, Düren, Germany), according to the manufacturer’s instructions. cDna was synthesized using High-Capacity cDNA Reverse Transcription Kit (Applied Biosystems) from 4 µg total RNA. The applied PCR settings were previously described^45^. The used gene primers were: *Il1b* (for IL-1β)*, Il6* (for IL-6)*, Tnfa* (for TNF-α)*, Prostaglandin-endoperoxide synthase* (*Ptgs) 2* (for COX-2), *Ifng* (for IFN-γ), *AnxA1* (for Annexin A1)*, Il10* (for IL-10)*, Tff3* (for TFF3), *Muc2* (for mucin 2), *Ocln* (for Occludin)*, Tlr2* (for TLR2)*, MyD88* (for MYD88)*, Hdac9* (for HDAC9), *Slc16a1* (for MCT1) and *Nlrp3* (for NALP3) (Qiagen, Hilden, Germany) in a final volume of 25 μl. All studied mRNAs were normalized to *Gapdh* (for GAPDH) or *Actb* (for β-actin) as housekeeping gene, and data were analyzed according to the 2^−ΔΔCT^ method.

### Histologic analysis of the colon

At 15 d, colon tissues were collected and stored in formalin 10%. Hematoxylin and eosin staining (H&E) was performed and colon sections were analyzed by the same pathologist in a blinded manner to evaluate their structure and architecture. Histopathology was quantified based on the scoring system indicating mucus thickness and the presence of apoptotic bodies for each animal. Morphological changes were evaluated in comparison with CON section.

### Western Blot analysis

Colon and liver tissues were homogenized and total protein lysates were subjected to SDS-PAGE. To evaluate NFκB activation and histone H3 acetylation, NFκB p65 (Cell Signaling Technology), Acetyl-H3 and H3 (EMD Millipore), and IκBα were measured in nuclear and cytosolic extracts, respectively, as previously described^46^. Blots were probed with anti-aquaporin (AQP) 9 (Biorbyt), GPR43 (EMD Millipore), TRL4 (Santa Cruz Biotechnology). Western blot for lamin-A, β-Actin and GAPDH (Sigma-Aldrich) was performed to ensure equal sample loading in nuclear and total lysates, respectively.

### Microbiota sequencing and data analysis

A subset of mice from each group was analyzed for gut microbiota. Fecal microbiota of control (CON) mice treated with vehicle for 5 or 15 days (CON 5 and CON 15, respectively) and of AIJ 5, AIJ 5 + FBA, AIJ 15, AIJ 15 + FBA groups was analyzed. Mice were housed in the same room, 2 per cage, sorted by treatment and were from different litters. Bacterial genomic DNA was extracted from frozen fecal samples using the QIAamp DNA Stool Mini Kit (Qiagen) according to manufacturer’s instructions. The quantity and quality of DNA was determined by spectrophotometric measurements (NanoDrop; Thermo Fisher Scientific Inc., Waltham, MA, USA). The V3-V4 region of the 16 S rRNA gene from each DNA sample was amplified and prepared for sequencing according to the protocol 16 S Metagenomic Sequencing Library Preparation for Illumina Miseq System. Barcoded amplicons were mixed in equal amounts based on concentrations as determined by Qubit Fluorometer (Invitrogen; Thermo Fisher Scientific, Inc.) and library sizes as assessed using a Bioanalyzer DNA 1000 chip (Agilent Technologies Gmbh, Waldbronn, Germany). Normalized libraries (4 nM) were pooled, denatured, diluted to 10 pM and combined with 25% (v/v) denatured 10 pM PhiX (Illumina, Inc.), according to Illumina guidelines. Sequencing run was performed on the Illumina MiSeq system using v3 reagents for 2 × 281 cycles (Illumina, Inc.).

V3-V4 16 S rDNA FASTQ paired-end reads were pre-processed with PEAR in order to retain high quality sequences with an overlap of at least 40 nucleotides, length comprised between 400 and 500 bp and with a PHRED score ≥ 33. These reads were then processed with PRINSEQ to obtain FASTA and QUAL files for further analyses. Pick of operational taxonomic units (OTUs), taxonomic assignment and diversity analyses were conducted using Quantitative Insights Into Microbial Ecology (QIIME) (version 1.8.0). A closed reference-based OTU picking method was employed to obtain OTUs at 97% sequence similarity from (May 2013 version). The Greengenes 16S gene database (GG) database was used to taxonomically classify the identified OTUs and to compute their distribution across different taxonomic levels. Samples were normalized to 23,422 sequences/sample using a sequence rarefaction procedure. Species heterogeneity within each sample was assessed by employing two Alpha diversity metrics (the number Observed species and the Shannon entropy) and compared using a two-sample permutation t-test, using 999 Monte Carlo permutations to compute p-values. OTUs diversity among microbial communities (beta diversity) was assessed by calculating unweighted Unifrac distances and compared by using ANOSIM method with 999 permutations. One-way ANOVA followed by Tukey multiple comparison post-hoc tests were used to determine statistical differences in OTUs frequencies among groups across different taxonomic levels. Species classification was performed using SPecies IdentificatioN of metaGenOmic amplicons program (SPINGO) (version 1.3) with default parameters on a representative sequence of each OTU. Metagenomes were predicted using PICRUSt based on normalized OTU table, corrected for multiple 16S rRNA gene copy number. Kyoto encyclopedia of genes and genomes (KEGG) ortholog abundances of key functions involved in butyrate metabolism among groups were compared using one-way ANOVA followed by Tukey multiple comparison post-hoc tests. Pearson correlation test was used to assess the eventual association between the amount of key bacterial species and KEGG ortholog counts of key functions involved in butyrate metabolism.

### Statistical analysis

All shown values were indicated as means ± SEM and statistical significance was set at *P* < 0.05. The statistical analyses were performed with the use of Graph-Pad Prism 7 (Graph-Pad Software, La Jolla, CA). For all the experimental data, differences among groups were compared by ANOVA, followed by Bonferroni multiple comparison test for multiple comparisons.

## Supplementary information

Supplementary info
