# Supplementary material for: N-(1-carbamoyl-2-phenylethyl) butyramide reduces antibiotic-induced intestinal injury, innate immune activation and modulates microbiota composition
Source: Sci Rep. 2019 Mar 18;9:4832. doi: 10.1038/s41598-019-41295-x (PMC6423286; doi:10.1038/s41598-019-41295-x)
Supplement: Supplementary file 1 — Supplementary info [file 41598_2019_41295_MOESM1_ESM.pdf]

N-(1-carbamoyl-2-phenylethyl) butyramide reduces antibiotic-induced intestinal injury, innate immune activation and modulates microbiota composition

Adriano Lama[1,2], Chiara Annunziata[1], Lorena Coretti[2,3], Claudio Pirozzi[1], Francesca Di Guida[1], Allegra Nitrato Izzo[1], Claudia Cristiano[1,2], Maria Pina Mollica[2,4], Lorenzo Chiariotti[2,3,5], Alessandra Pelagalli[6,7], Francesca Lembo[1,2], Rosaria Meli[1] and Giuseppina Mattace Raso[1,2].

[1]Department of Pharmacy, [2]Task Force on Microbiome Studies, [4]Department of Biology, [5]Department of Molecular Medicine and Medical Biotechnology, [6]Department of Advanced Biomedical Sciences, University of Naples "Federico II", 80131 Naples, Italy; [3]Institute for Experimental Endocrinology and Oncology, IEOS, [7]Institute of Biostructure and Bioimaging, Consiglio Nazionale delle Ricerche CNR, Via S. Pansini, 5, 80131, Naples, Italy.

Corresponding author: Rosaria Meli, via D. Montesano, 49-80131, Naples, Italy, +39-081-678413, fax +39-081-678403; E-mail: meli@unina.it.

Supplementary tables

Table S1. Alpha-diversity indexes of CON, AIJ and AIJ + FBA microbial communities at 5 and 15 days. Data are mean ± SD; different letter indicated significant differences among groups ($p < 0.05$) as assessed by one-way ANOVA followed by Tukey's multiple comparison post-hoc test.

α-index	CON 5	AIJ 5	AIJ 5 + FBA	CON 15	AIJ 15	AIJ 15 + FBA
Good's coverage	0.99 ± 1E-3[a]	0.99 ± 3E-4[a]	0.99 ± 2E-3[a]	0.99 ± 4E-4[a]	0.99 ± 4E-4[a]	0.99 ± 1E-3[a]
Observed species	827.6 ± 88.3[a]	403.7 ± 32.9[b]	523 ± 127.8[c]	866.7 ± 29[a]	328.5 ± 32.9[b]	218.5 ± 20.2[d]
Shannon index	6.5 ± 0.4[a]	3.8 ± 0.7[a]	4.1 ± 0.4[a]	6.9 ± 0.1[a]	4 ± 0.1[a]	4.1 ± 0.4[a]

Table S2. Gut microbiota structure at phylum level of CON, AIJ and AIJ + FBA microbial communities at 5 and 15 days. Data are mean relative abundance ± SEM; different letters indicated significant differences among groups ($p < 0.05$) as assessed by one-way ANOVA followed by Tukey's multiple comparison *post-hoc* test.

Bacterial phylum	CON 5	AIJ 5	AIJ 5 + FBA	CON 15	AIJ 15	AIJ 15 + FBA
Acidobacteria	0[a]	0.09 ± 0.07[a]	0.03 ± 0.01[a]	0[a]	0[a]	0[a]
Actinobacteria	0.04 ± 0.01[a]	3.76 ± 0.44[a]	5.85 ± 1.30[a]	0.03 ± 0.01[a]	0.28 ± 0.08[a]	0.09 ± 0.04[a]
Bacteroidetes	57.12 ± 9.85[a]	1.51 ± 0.11[b]	2.05 ± 0.60[b]	55.49 ± 1.53[a]	39.83 ± 7.43[c]	0.19 ± 0.06[b]
Chloroflexi	0[a]	0.01 ± 0.01[a]	3E-3 ± 2E-3[a]	0[a]	0[a]	0[a]
Cyanobacteria	0.48 ± 0.37 [a]	62.26 ± 11.11[b]	54.33 ± 13.10[b]	0.39 ± 0.14[a]	0.02 ± 4E-3[a]	0.12 ± 0.02[a]
Deferribacteres	1E-3 ± 1E-3[a]	0[a]	0.03 ± 0.02[a]	0.02 ± 0.01[a]	0[a]	0[a]
Firmicutes	39.36 ± 10.26[a]	4.519 ± 0.40[b]	7.96 ± 1.68[b]	32.30 ± 0.71[a]	43.28 ± 7.10[a]	90.0 ± 5.80[c]
Fusobacteria	0[a]	4E-3 ± 4E-3[a]	0.05 ± 0.02[a]	0[a]	0[a]	0[a]
GN02	0[a]	0[a]	0.12 ± 0.07[a]	0[a]	0[a]	0[a]
Gemmatimonadetes	0[a]	0[a]	0.02 ± 4E-3 [a]	0[a]	0[a]	0[a]
OD1	0[a]	0[a]	0.03 ± 0.02[a]	0[a]	0[a]	0[a]
Planctomycetes	0[a]	0[a]	0.01 ± 3E-3 [a]	0[a]	0[a]	0[a]
Proteobacteria	2.41 ± 0.06[a]	27.689 ± 11.795[b]	29.376 ± 16.371[b]	7.161 ± 0.433[a]	0.013 ± 0.006[a]	0.116 ± 0.01[a]
TM7	0.339 ± 0.065[a]	0.028 ± 0.022[a]	0.004 ± 0.004[a]	0.266 ± 0.052[a]	0[a]	0[a]
Tenericutes	0.199 ± 0.1[a]	0[a]	0[a]	0.111 ± 0.044[a]	2.079 ± 0.204[a]	0.001 ± 0.001[a]
Verrucomicrobia	0.044 ± 0.044[a]	0.116 ± 0.076[a]	0.158 ± 0.066[a]	4.235 ± 0.488[ab]	14.513 ± 0.61[b]	9.487 ± 5.785[ab]
WPS-2	0[a]	0.011 ± 0.011[a]	0.009 ± 0.009[a]	0[a]	0[a]	0[a]
[Thermi]	0[a]	0[a]	0.002 ± 0.002[a]	0[a]	0[a]	0[a]

Table S3. KEGG Orthologs count of genes codifying for key enzymes involved in butyrate metabolism. Data are means ± SEM; different letter indicated significant differences among groups ($p < 0.05$) as assessed by one-way ANOVA followed by Tukey's multiple comparison post-hoc test.

KEGG Ortholog: Description	CON 5	AIJ 5	AIJ 5 + FBA	CON 15	AIJ 15	AIJ 15 + FBA
K00626: acetyl-CoA C-acetyltransferase	3297.3 ± 832.5[a]	10283.5 ± 4385.4[a]	8004.5 ± 2497.0[a]	3182.0 ± 136.2[a]	7130.7 ± 1535.3[a]	7236 ± 310.6[a]
K00074: 3-hydroxybutyryl-CoA dehydrogenase	3271.3 ± 924.9[a]	2975.7 ± 973.6[a]	2248.0 ± 316.3[a]	3232.7 ± 103.9[a]	4463.0 ± 231.8[a]	4673.0 ± 326.5[a]
K01692: enoyl-CoA hydratase	913.3 ± 323.0[a]	23715.0 ± 5726.7[b]	17668.0 ± 1375.4[b]	1096.0 ± 47.7[a]	1679.2 ± 710.3[a]	721.0 ± 239.9[a]
K01715: 3-hydroxybutyryl-CoA dehydratase	2784.3 ± 757.5[a]	542.0 ± 96.0[b]	800.2 ± 150.4[bc]	2230.0 ± 54.4[ac]	2274.7 ± 407.9[ac]	3888.7 ± 531.1[a]
K00248: butyryl-CoA dehydrogenase	3076.3 ± 890.5[abc]	2071.2 ± 814.4[ac]	1136.2 ± 120.2[c]	2621.2 ± 68.7[ac]	4459.7 ± 1067.6[ab]	5731.2 ± 224.9[b]
K00634: phosphate butyryltransferase	6337.7 ± 314.1[a]	181.0 ± 16.6[b]	243.5 ± 73.0[b]	5652.0 ± 124.3[ac]	5431.0 ± 136.1[ac]	4503.2 ± 596.8[c]
K00929: butyrate kinase	6783.0 ± 83.9[ac]	224.2 ± 31.6[b]	291.7 ± 81.4[b]	5833.0 ± 131.5[ac]	7171.0 ± 695.6[a]	4946.5 ± 675.5[c]
K01034/5: acetate CoA-transferase alpha/beta subunit	273.0 ± 167.9[ab]	98.7 ± 44.5[a]	78.2 ± 30.5[a]	423.7 ± 75.4[ac]	717.7 ± 31.8[bc]	2017.2 ± 223.3[d]
K00016: L-lactate dehydrogenase	3146.3 ± 804.6[ac]	1026.7 ± 142.3[a]	1538.0 ± 324.3[a]	2561.0 ± 53.2[ac]	4087.2 ± 739.0[c]	7384.5 ± 548.6[b]

Figure S1. Effect of FBA on serum parameters. (A-G) TNF-α, IL-1β, IFN-γ, LDH, IL-10, AST, and ALT are reported (*n*=6). Data are mean ± S.E.M. All results were considered statistically significant at *P*<0.05. Labeled means without a common letter differ, *P*<0.05.

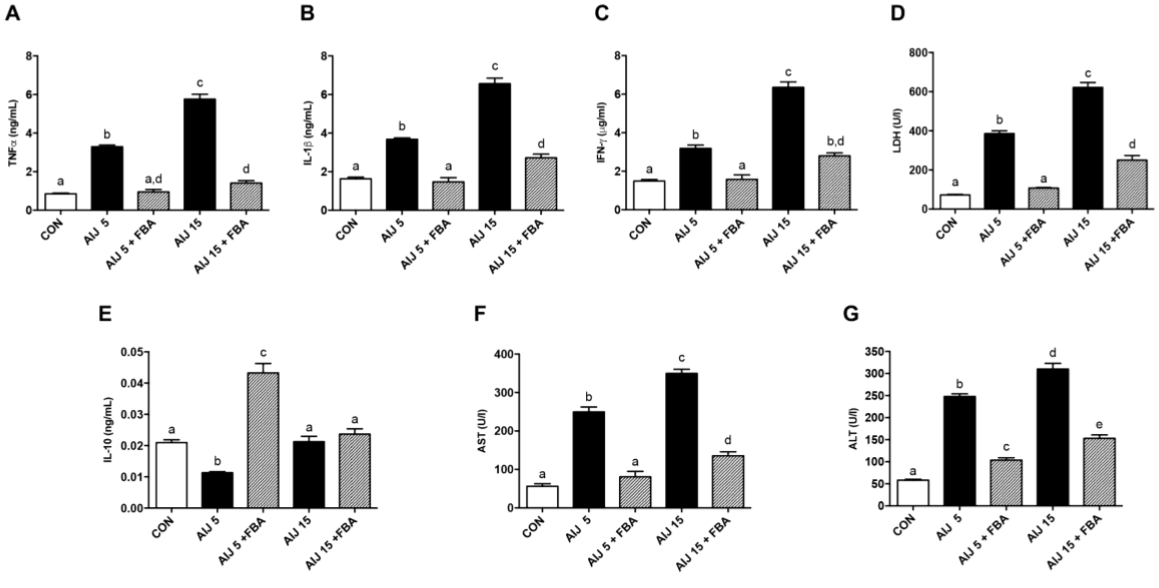

Figure S2. Original blots of manuscript.

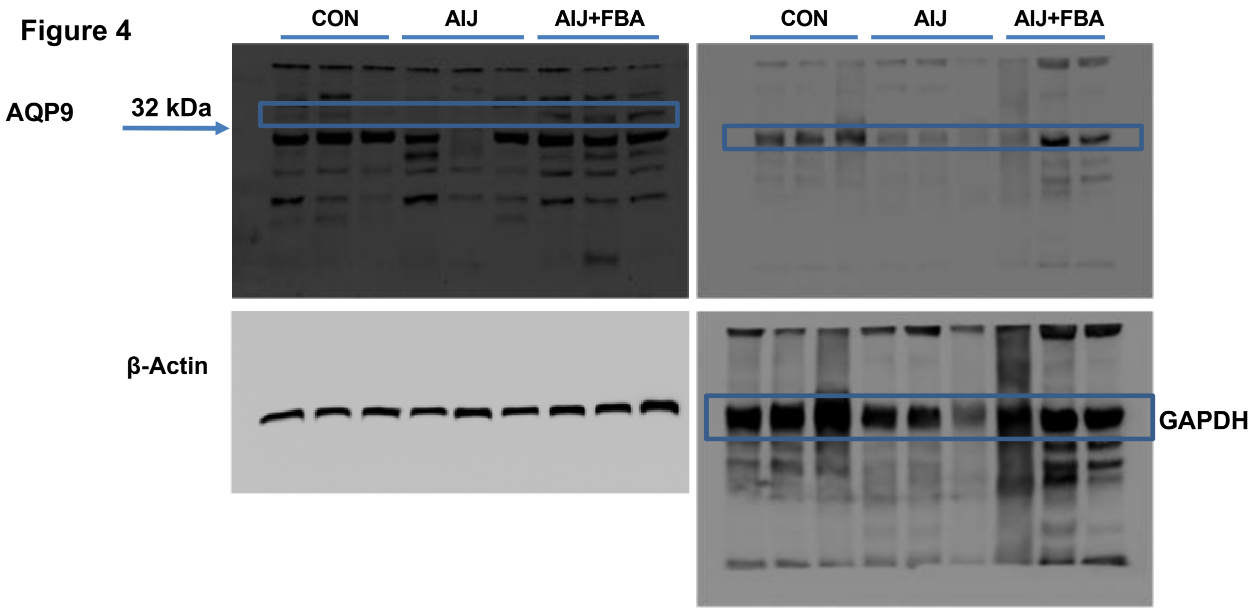

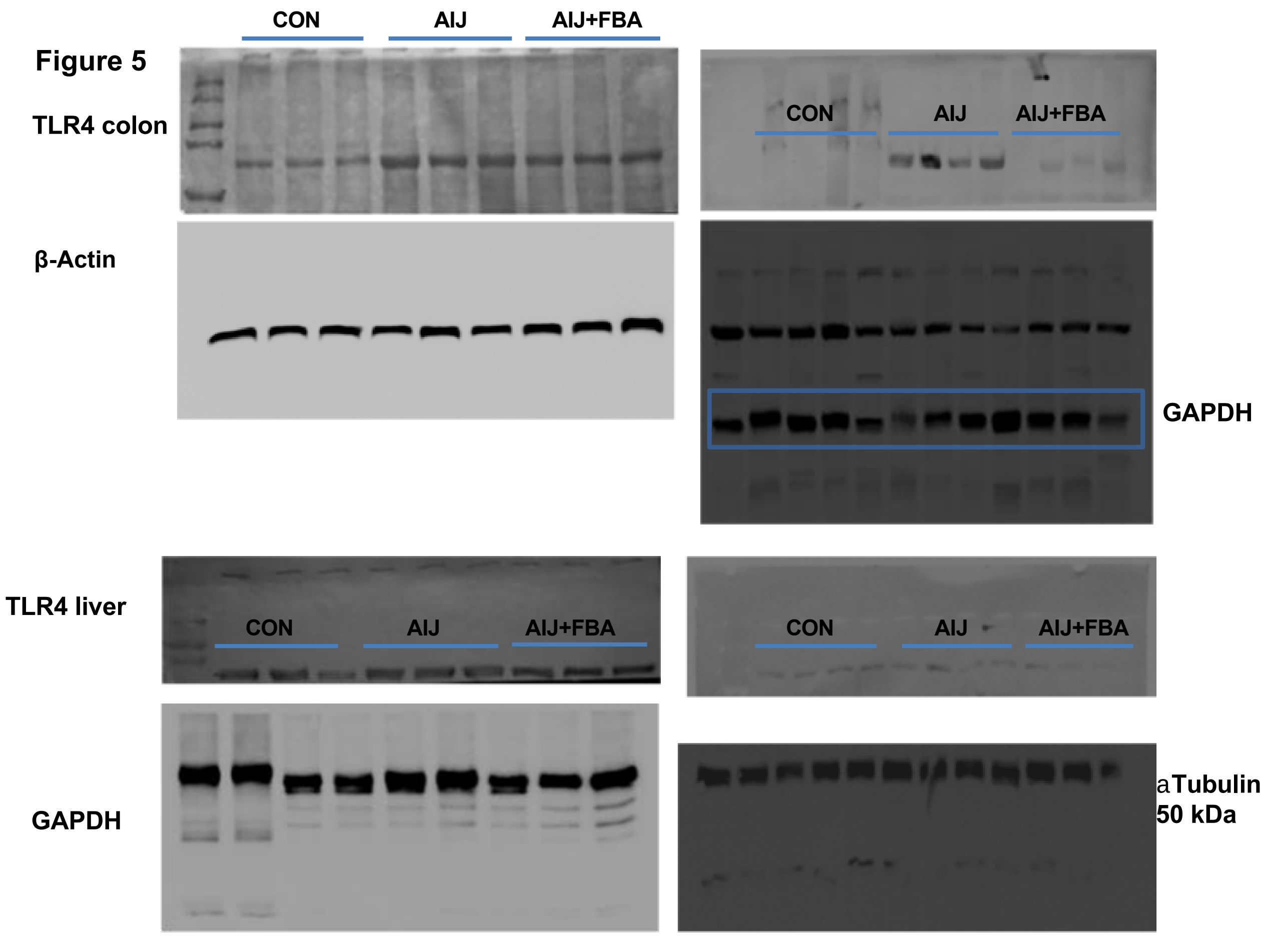

Figure 5

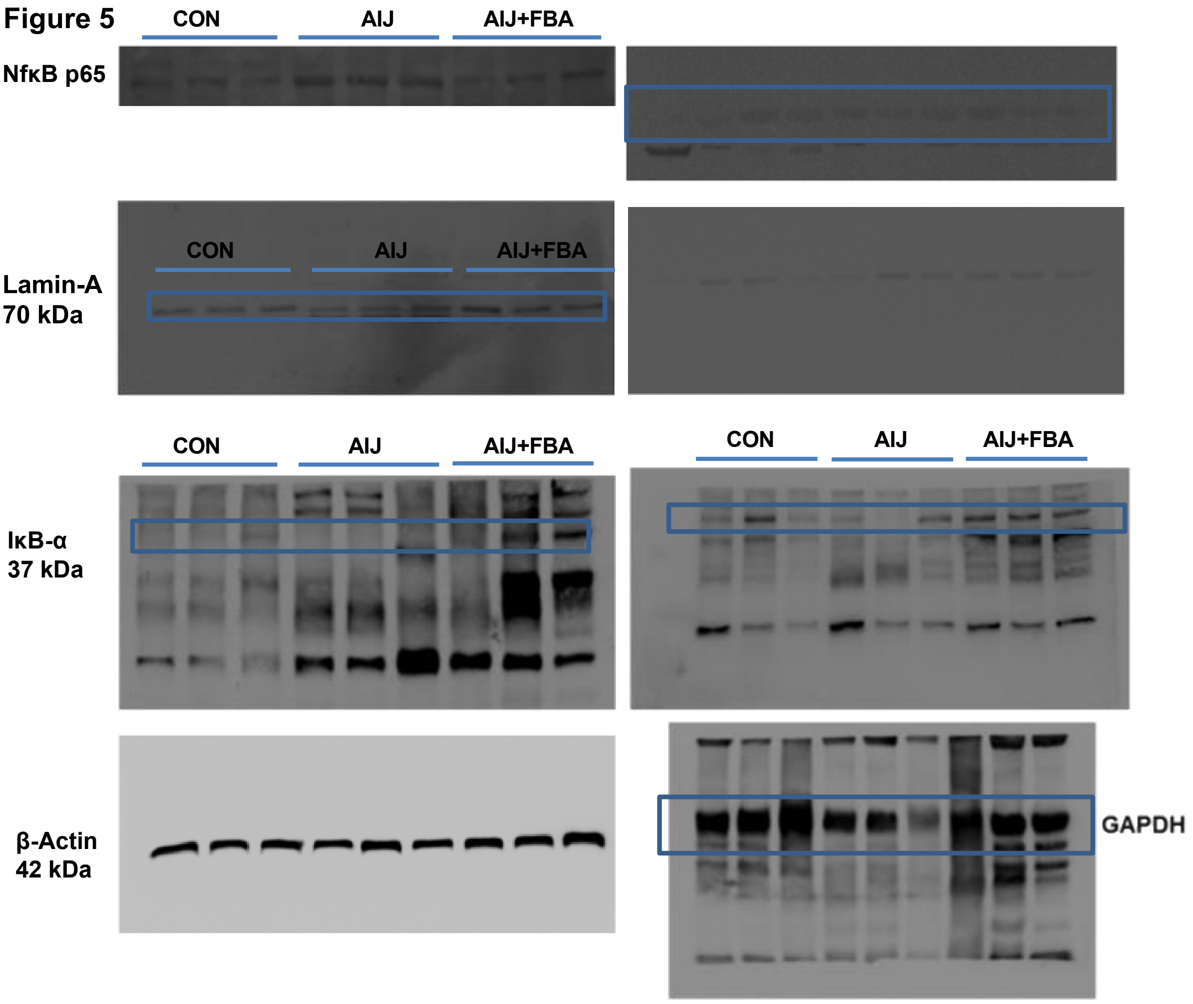
CON
AIJ
AIJ+FBA
NfκB p65
CON
AIJ
AIJ+FBA
Lamin-A
70 kDa
CON
AIJ
AIJ+FBA
CON
AIJ
AIJ+FBA
IκB-α
37 kDa
β-Actin
42 kDa
GAPDH

Figure 6

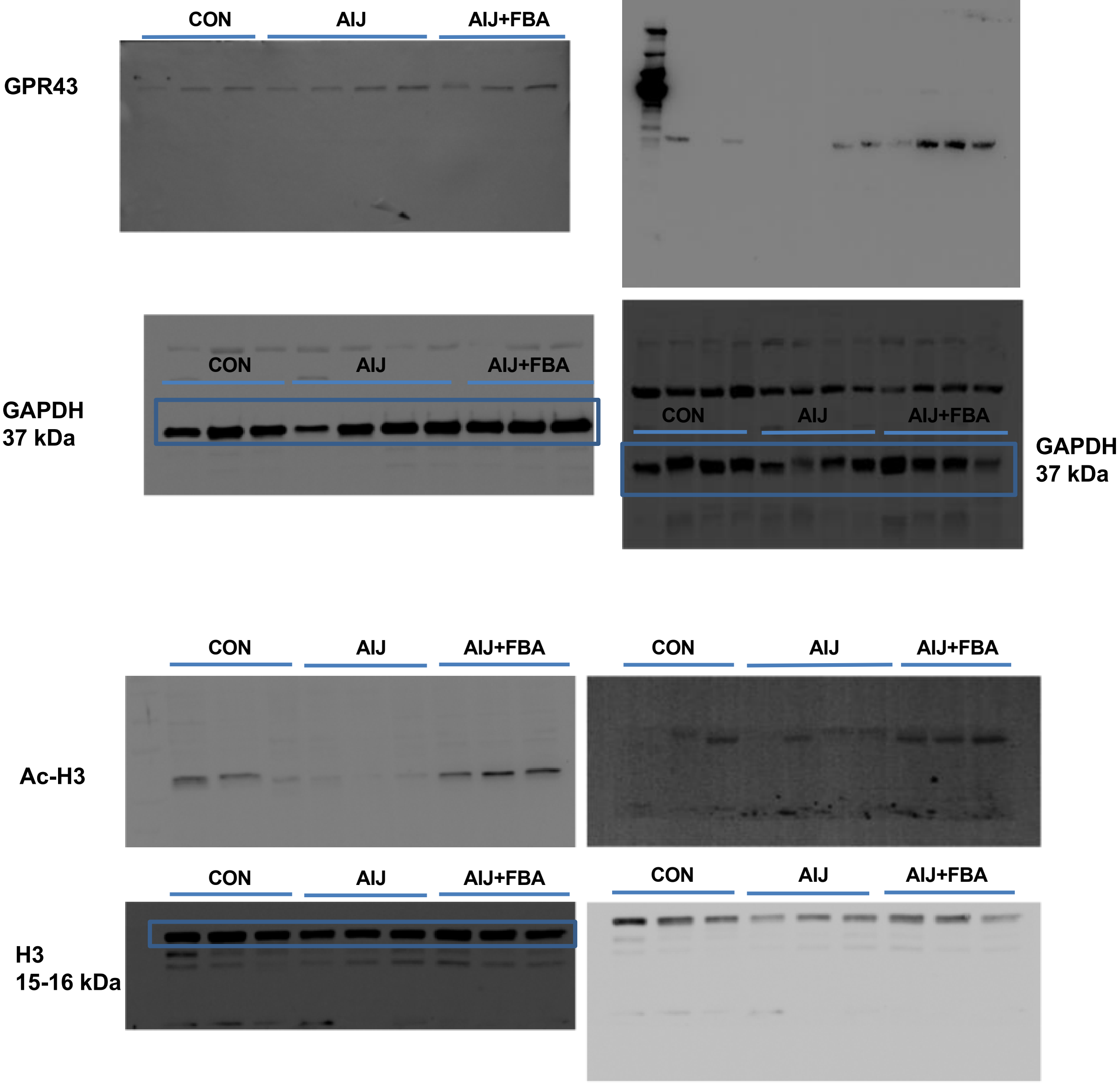

CON
AIJ
AIJ+FBA
GPR43
CON
AIJ
AIJ+FBA
GAPDH
37 kDa
CON
AIJ
AIJ+FBA
GAPDH
37 kDa
CON
AIJ
AIJ+FBA
CON
AIJ
AIJ+FBA
Ac-H3
CON
AIJ
AIJ+FBA
CON
AIJ
AIJ+FBA
H3
15-16 kDa
